# Oestrogen receptor **β** expression and depth of myometrial invasion in human endometrial cancer

**DOI:** 10.1054/bjoc.2000.1589

**Published:** 2001-02

**Authors:** F Takama, T Kanuma, D Wang, I Kagami, H Mizunuma

**Affiliations:** Department of Obstetrics and Gynaecology, Gunma University School of Medicine, 3-39-22 Showamachi, Maebashi, Gunma 371 8511, Japan

**Keywords:** endometrial cancer, oestrogen receptor alpha and beta, myometrial invasion

## Abstract

We assessed the relative expression of oestrogen receptor (ER)α and oestrogen receptor (ER)β mRNAs in 36 human endometrial cancers using a multiplex polymerase chain reaction (PCR). To determine whether or not the expression of ER subtypes in endometrial cancers is associated with clinicopathological parameters, we examined correlations between ER subtypes and age, tumour grade and depth of myometrial invasion. Using multiple regression analysis, myometrial invasion showed a significant correlation with ER-β: ER-α ratio (r = 0.54, *P* = 0.0007). The ER-β:ER-α ratio was high in advanced invasive carcinoma. Western blotting analysis showed that ER-β proteins were highly expressed in comparison with ER-α proteins in endometrial cancer with severe myometrial invasion. Our results suggest that ER-β is important in the progression of myometrial invasion. © 2001 Cancer Research Campaign http://www.bjcancer.com


*British Journal of Cancer* (2001) **84**(4), 545–549
© 2001 Cancer Research Campaign
doi: 10.1054/ bjoc.2000.1589, available online at http://www.idealibrary.com on IDEAL®

### Oestrogen receptor β expression and depth of myometrial invasion in human endometrial cancer

**F Takama, T Kanuma, D Wang, I Kagami and H Mizunuma**

Department of Obstetrics and Gynaecology, Gunma University School of Medicine, 3-39-22 Showamachi, Maebashi, Gunma 371 8511, Japan

**Summary** We assessed the relative expression of oestrogen receptor (ER)α and oestrogen receptor (ER)β mRNAs in 36 human endometrial cancers using a multiplex polymerase chain reaction (PCR). To determine whether or not the expression of ER subtypes in endometrial cancers is associated with clinicopathological parameters, we examined correlations between ER subtypes and age, tumour grade and depth of myometrial invasion. Using multiple regression analysis, myometrial invasion showed a significant correlation with ER-β: ER-α ratio ($r = 0.54$, $P = 0.0007$). The ER-β:ER-α ratio was high in advanced invasive carcinoma. Western blotting analysis showed that ER-β proteins were highly expressed in comparison with ER-α proteins in endometrial cancer with severe myometrial invasion. Our results suggest that ER-β is important in the progression of myometrial invasion. © 2001 Cancer Research Campaign http://www.bjcancer.com

**Keywords:** endometrial cancer; oestrogen receptor alpha and beta; myometrial invasion

Oestrogen receptors (ER) are encoded by the ER-α and ER-β genes (Kuiper et al, 1996; Mosselman et al, 1996). These two receptors share about 95% homology in the DNA binding domain and 55% homology in the ligand binding domain, bind to a consensus oestrogen response element (ERE) (Tremblay et al, 1997) and have similar ligand binding properties (Kuiper et al, 1997). The reverse transcription polymerase chain reaction (RT-PCR) has revealed that ER-β is expressed at high levels in the prostate and ovary (Kuiper et al, 1996; Mosselman et al, 1996), and at moderate levels in many other tissues, including the testis and the uterus, parts of which also seem to express ER-β (Kuiper et al, 1997). Oestrogen promotes the progression of breast cancer. The presence of ER-α and ER-β mRNA in both normal and neoplastic human breast tissues has been reported (Dotzlaw et al, 1997). Furthermore, the relative expression of ER-α and ER-β mRNA differs between normal human breast tissues and concurrently matched ER-positive breast tumours (Leygue et al, 1998), suggesting that ER-α and ER-β expression is functionally altered during breast tumorigenesis. Although the two receptors are often coexpressed in breast cancer, the levels of ER-β mRNA appear to vary among breast tumours (Dotzlaw et al, 1997), raising the question as to whether ER-β expression correlates with prognostic or endocrinological markers. The expression of ER-β mRNA is inversely correlated with progesterone receptor (PR) status, suggesting that ER-β expression is regulated by progestins (Dotzlaw et al, 1999). However, no specific data regarding ER- β distribution in endometrial cancer have been published. Therefore, we assessed the relative expression of ER-α and ER-β mRNAs in endometrial cancers, to determine whether or not the expression of these receptors is altered during endometrial tumorigenesis.

*Received 11 May 2000*
*Revised 19 September 2000*
*Accepted 18 October 2000*

*Correspondence to:* H Mizunuma

### MATERIAL AND METHODS

### Tissues and RNA extraction

We examined samples of 35 endometrial cancers, 1 of complex hyperplasia and 1 normal endometrium in the secretory phase obtained with consent from patients who were treated at the Gunma University Hospital, Department of Obstetrics and Gynaecology. Samples were immediately frozen in liquid nitrogen and stored at –80˚C. No chemotherapy or radiation therapies were performed prior to tumour excision. Surgically resected tissues were sampled for histopathological diagnosis. Clinical stage and histological classification were established according to the International Federation of Gynaecology and Obstetrics (FIGO) 1988 criteria. Myometrial invasion was classified into 7 grades. Grade 0 indicates a lack of myometrial invasion. Depth of myometrial invasion was measured microscopically and categorized from grades 1/6 to 6/6, 6/6 means carcinoma reached the serosa. Details are presented in Tables 1 and 2. Total RNA was extracted from frozen tissue sections using the GLASS MAX RNA Microisolation Spin Cartridge System (Gibco/BRL, Grand Island, NY) according to the manufacturer's instructions.

### Primers and RT-PCR conditions

Total RNA (1 μg) was reverse transcribed in 20 μl of reaction mix for 59 min at 42˚C, then for 15 min at 70˚C. The reaction mix contained 50 units of Moloney murine leukaemia virus reverse transcriptase (Perkin Elmer, Foster City, CA), 0.8 mM dNTPs (Perkin Elmer), 20 units of RNase inhibitor (Perkin Elmer), 10 mM Tris-HCl (pH8.3), 50 mM KCl, 1.5 mM $MgCl_2$ and 2.5 μM random hexamers (Perkin Elmer). To determine the relative expression of ERα and ERβ mRNA within individual samples, two sets of primers were added to each reaction and both ERα and ERβ cDNAs were co-amplified. Reverse transcription reaction (3 μg) was amplified by PCR in a final volume of 25 μl, containing 1 unit of Ampli Taq Gold (Perkin Elmer), 10 mM Tris-HCl (pH 8.3), 50 mM KCl, 1.5 mM $MgCl_2$, 0.2 mM dNTPs, and 2 μM of

Table 1 Pathologic details of endometrial cancers used in this study

| Clinical parameters | | No. |
|---|---|---|
| Patients | Total no. in study | 36 |
| | Mean age (range) | 56 (36~81) |
| | <50 years | 10 |
| | ≧50 years | 26 |
| Tumour histology | endometrioid | 32 |
| | clear cell | 1 |
| | adenosquamous | 1 |
| | serous | 1 |
| | complex hyperplasia | 1 |
| Tumour grade | Grade 1 | 19 |
| | Grade 2 | 9 |
| | Grade 3 | 4 |
| | Others | 4 |
| Myometrial invasion | 0 | 1 |
| | 1/6 | 19 |
| | 2/6 | 7 |
| | 3/6 | 3 |
| | 4/6 | 2 |
| | 5/6 | 1 |
| | 6/6 | 3 |
| Clinical stage | Ia | 0 |
| | b | 22 |
| | c | 3 |
| | IIa | 4 |
| | b | 2 |
| | IIIa | 2 |
| | b | 0 |
| | c | 1 |
| | IV | 1 |

each primer under the following conditions: 95˚C for 10 min followed by 30 cycles at 94˚C for 30 sec and 72˚C for 3 min and an extension step at 72˚C 5 min. Under these conditions, PCR products were generated within a linear range. Primers used to amplify ER-α were: (sense) 5′-CTGGCTACATCATCTCGGTTCCGCA-3′ (nucleotides 1577~1601); (antisense) 5′-TCAGGCCTGCCTTG-GCCATCAGGT-3′ (nucleotides 1755~1778), generating an amplified product of 202 bp. For ER-β, primer sequences were: (sense) 5′- GCGCTCAATTGACCACCCCGGCAA-3′ (nucleotide 894~927); (antisense) 5′-GCATCGGTCACGGCGTTCAGCAAG-3′ (nucleotide 1143~1166), generating an amplified product of 273 bp. The ubiquitously expressed GAPDH cDNA was amplified in parallel, to prove that used RNAs were not degraded.

PCR products were subcloned into the pGEM-T Easy vector (Promega, Madison, WI) and sequenced using a cycle sequencing kit (Takara Shuzo; Shiga, Japan).

### Multiplex PCR validation

Two tumours (T1 and T2) were used to validate a multiplex RT-PCR that was designed to determine the relative expression of ER-β: ER-α. T1 expressed medium ER-β/high ER-α mRNA levels, on the other hand, T2 expressed low ER-β/high ER-α mRNA levels. 6 cDNA preparations were prepared containing varying percentages of T1 and T2 cDNA by mixing 5, 4, 3, 2, 1 and 0 μl of T1 cDNA with 0, 1, 2, 3, 4 and 5 μl of T2 cDNA (100, 80, 60, 40, 20 and 0% T1 cDNA, respectively). The two receptor subtypes were coamplified in 3 μl of cDNA preparation as described above. PCR products were separated on 1.8% Metaphor agarose gels (FMC Bio Products; Rockland, USA) and visualized by ethidium bromide staining under UV illumination.

### Quantitation and statistical analysis

To compare ER-β mRNA expression relative to that of ER-α mRNAs, ER-α and ER-β cDNA were coamplified as described above. Images were captured under UV transillumination on type 667 film (Polaroid Co, Cambridge, MA) and signals were quantified using BAS-2000 imaging analyser (Fuji Photo Film Co, Tokyo Japan). Statistical analysis was performed using the Stat Flex for Windows (Artech Inc, Osaka, Japan). Multiple regression method was used to analyse the association between the β:α ratio and clinical factors including age, tumour grade, clinical stage and myometrial invasion.

### Western blot analysis

Of 37 endometrial tissue samples, 34 were analysed by Western blotting. The samples were homogenized in 1 × SDS buffer (2% SDS, 100 mM DTT and 60 mM Tris-HCl pH 6.8). Then, the preparations were boiled for 5 min, and passed through a 26G needle with a 1 ml syringe. After monitoring at OD 280/260, they were diluted with 1 × SDS buffer, and 0.4 units (OD280 = 1 unit) of each sample was applied to SDS-PAGE. Gels were soaked in transfer buffer (48 mM Tris-HCl, 30 mM glycine and 20% methanol, pH 9.2) and proteins were transferred to nitrocellulose membranes (Hybond ECL, Buckinghamshire, England). Immunoblotting was performed using anti-ER-α, anti-ER- β, anti-PR (Santa Cruz Biotechnology; Santa Cruz, CA) and anti-β-actin (SIGMA; Japan). Signals were developed using ECL Western blotting detection reagents (Amersham Pharmacia Biotech UK Limited).

### RESULTS

### Multiplex PCR validation

The relative expression of ER-α and ER-β mRNA within individual samples was determined using a multiplex PCR assay. Two sets of primers were added to each PCR mixture and both ER-α and ER-β were amplified in a single tube. To determine whether or not the results obtained from this assay accurately reflected the initial ER-β:ER-α ratio, a preliminary multiplex PCR was performed on tumours T1 and T2. Tumour T1 expressed medium ER-β/high ER-α mRNA levels, whereas T2 expressed low ER-β/high ER-α mRNA levels. As shown in Figure 1A, the PCR signal corresponding to ER-β decreased with decreasing input of T2 cDNA, and the ER-α signal increased with increasing input of T1 cDNA. The ER-β:ER-α ratio signals were plotted as a function of the percentage of T1 cDNA input. The ER-β:ER-α ratio increased in a linear fashion as the ER-β input increased. Multiplex PCR under these conditions showed a dose related increase until the relative expression of ER-β and ER-α was <0.5 (Figure 1B).

### Expression of ER-α and ER-β mRNAs in human endometrial cancer tissues, and association with clinical information

35 endometrial cancer samples, 1 of complex hyperplasia and 1 normal endometrium were analysed and the expression of both ER-α and ER-β was compared by multiplex PCR (Figure 2). The 202 bp and 273 bp DNA fragments from a tumour sample that emitted intense ER-α and ER-β signals were subcloned and

 © 2001 Cancer Research Campaign

**Table 2** Clinical characteristics of patients in this study

| Case | Age | Histology | Clinical stage | Histological grade | Myometrial invasion | Surgical procedure | Adjuvant therapy | Outcome | β/α ratio ×100 |
|---|---|---|---|---|---|---|---|---|---|
| 1 | 58 | endometrioid | Ib | G1 | 2/6 | mRH,BSO,PLA | CAP2 | death(3mo)*[1] | 7.7 |
| 2 | 50 | endometioid | Ib | G1 | 1/6 | mRH,BSO,PLA | | dfs(2y7mo) | 0.0 |
| 3 | 81 | endometrioid | IIa | G3 | 2/6 | ATH,BSO | | death(2y5mo) | 2.5 |
| 4 | 48 | endometrioid | IIa | G2 | 1/6 | ARH,BSO,PLA | | dfs(2y9mo) | 7.6 |
| 5 | 58 | endometrioid | Ib | G3 | 1/6 | mRH,BSO,PLA | CAP3 | dfs(2y5mo) | 11.0 |
| 6 | 80 | clear cell | Ic | | 5/6 | ATH,BSO | | dfs(2y8mo) | 47 |
| 7 | 44 | adenosquamous | Ib | | 1/6 | mRH,BSO,PLA,PAN | | dfs(2y6mo) | 6.8 |
| 8 | 60 | endometrioid | Ib | G3 | 1/6 | ATH,BSO | CAP3 | dfs(2y8mo) | 0.0 |
| 9 | 51 | endometrioid | Ib | G2 | 1/6 | ATH,BSO | CAP5 | dfs(2y4mo) | 6.3 |
| 10 | 55 | endometrioid | Ib | G1 | 3/6 | mRH,BSO,PLA | | dfs(2y2mo) | 31.5 |
| 11 | 47 | endometrioid | Ib | G1 | 1/6 | mRH,BSO,PLA | | dfs(2y1mo) | 1.5 |
| 12 | 74 | serous adeno | IIIc | | 1/6 | mRH,BSO,PLA | radiation | death(1y6mo) | 6.0 |
| 13 | 52 | endometrioid | IIb | G1 | 2/6 | mRH,BSO,PLA | | dfs(2y0mo) | 5.6 |
| 14 | 45 | endometrioid | IIa | G1 | 2/6 | mRH,BSO,PLA | CAP3 | dfs(1y8mo) | 12.2 |
| 15 | 36 | endometrioid | Ib | G1 | 2/6 | ATH,BSO | | death(POD6)*[2] | 4.3 |
| 16 | 46 | endometrioid | Ib | G1 | 1/6 | mRH,BSO,PLA | CAP3 | dfs(1y7mo) | 8.2 |
| 17 | 69 | endometrioid | IIa | G2 | 6/6 | mRH,BSO,PLA | CAP3 | dfs(1y7mo) | 53.4 |
| 18 | 51 | endometrioid | Ib | G1 | 1/6 | mRH,BSO,PLA | CAP5 | dfs(1y5mo) | 14 |
| 19 | 50 | endometrioid | Ib | G2 | 3/6 | mRH,BSO,PLA | CAP | dfs(1y6mo) | 19 |
| 20 | 50 | endometrioid | Ib | G1 | 1/6 | mRH,BSO,PLA | CAP3 | dfs(1y7mo) | 3.4 |
| 21 | 54 | endometrioid | Ib | G1 | 1/6 | mRH,BSO,PLA | | dfs(2y5mo) | 0.0 |
| 22 | 50 | endometrioid | Ib | G1 | 1/G | mRH,BSO,PLA | CAP4 | dfs(1y10mo) | 0.0 |
| 23 | 74 | endometrioid | IIIa | G1 | 6/6 | ATH,BSO | | dfs(2y6mo) | 16 |
| 24 | 53 | endometrioid | Ib | G1 | 1/6 | mRH,BSO,PLA | | dfs(1y6mo) | 1.5 |
| 25 | 47 | endometrioid | Ib | G2 | 2/6 | mRH,BSO,PLA | CAP3 | dfs(1y6mo) | 5.4 |
| 26 | 60 | endometrioid | Ib | G2 | 1/6 | ARH,PLA | CAP3 | dfs(1y5mo) | 11.1 |
| 27 | 58 | endometrioid | Ib | G1 | 1/6 | mRH,BSO,PLA | | dfs(1y5mo) | 9 |
| 28 | 65 | endometrioid | Ib | G1 | 1/6 | mRH,BSO,PLA | | dfs(1y4mo) | 16.4 |
| 29 | 70 | endometrioid | IV | G3 | 6/6 | probe lapa | CAP,TJ | death(2y5mo) | 31 |
| 30 | 53 | endometrioid | Ib | G2 | 4/6 | mRH,BSO,PLA | CAP | dfs(1y4mo) | 16.8 |
| 31 | 61 | endometrioid | Ib | G1 | 2/6 | mRH,BSO,PLA | | dfs(2y8mo) | 16.8 |
| 32 | 61 | endometrioid | Ic | G1 | 4/6 | ATH,BSO | | unknown | 29 |
| 33 | 41 | complex hyperplasia | | | p 0 | ATH,BSO | | dfs(3y8mo) | 1.2 |
| 34 | 49 | endometrioid | IIIa | G2 | 3/6 | mRH,BSO,PLA | CAP | dfs(2y6mo) | 4.5 |
| 35 | 41 | endometrioid | IIb | G1 | 2/6 | ARH,BSO,PLA | radiation | dfs(1y3mo) | 5 |
| 36 | 70 | endometrioid | Ic | G2 | 1/6 | mRH,BSO,PLA | | dfs(1y3mo) | 1.5 |
| 37 | 50 | normal(secretary phase) | | | | ATH | | | 14.6 |

endometrioid, endometrioid adenocarcinoma; adenosquamous, adenosquamous carcinoma; serous adeno, serous adenocarcinoma; clear cell, clear cell adenocarcinoma; mRH, modified radical hysterectomy; BSO, bilateral salpingo-oophorectomy; PLA, pelvic lymphadenectomy; PAN, para-aortic lymphadenectomy; ARH, abdominal radical hysterectomy; CAP, cyclophosphamide-farmorubicin-cisplatin; TJ, paclitaxel-carboplatin; dfs, desease free survival; pos, postoperative survival; *[1]Patient died of cerebral infarction, *[2]Patient died of pulmonary embolism.

sequenced. The tumour sequence was identical to that published for human ER-α and ER-β (Mosselman et al, 1996). ER-α was expressed constantly in all samples, whereas ER-β was expressed to varying degrees (Figure 2B). An ER-β:ER-α ratio of >0.5 was found in only one sample in the present study (Figure 2A). To determine whether or not ER subtype coexpression in endometrial cancers is associated with clinical parameters, we examined multiple regression analysis. Only myometrial invasion showed a significant correlation with the ER-β:ER-α ratio ($r = 0.54$, $P = 0.0007$) (Table 3). Figure 3 shows the correlation between the ER-β:ER-α ratio and depth of myometrial invasion. Spearman rank correlation test determined that the correlate coefficient was 0.786 ($P < 0.0001$). In one normal endometrium, the ER-β:ER-α ratio was in the medium range (0.145).

### Western blotting analysis

We examined the status of ER-α and ER-β protein by Western blotting in the 34 endometrial cancer tissue samples. The representative pattern of Western blotting was shown in Figure 2C. The relative expression of ER-β and ER-α protein was well correlated with analysis of mRNA. Among the 7 cases with severe myometrial invasion (depth ≥ 3/6), which expressed high ER-β:ER-β mRNA ratio (β/α ratio ≥ 0.16), 6 cases expressed ER-β protein relatively more strongly than ER-β protein (No. 6, 10, 17, 19, 23 and 30). Additionally, we analysed progesterone receptor (PR) protein expression, as some investigators have reported that expression of the progesterone receptor is related to good prognosis. PR protein was expressed in all endometrial cancer tissues to varying degrees, and was not correlated with depth of myometrial invasion (Figure 2C). There appeared to be no correlation between the PR protein expression pattern and clinicopathological parameters.

### DISCUSSION

Oestrogens are important mitogenic stimulants in endometrial and breast cancer in addition to playing important physiological roles in normal tissues. Oestrogen acts by binding to its specific receptors, ER-α and ER-β, although the physiological roles of these receptors have not been clearly distinguished. Dotzlaw et al (1997) showed that ER-α expression does not correlate with that of ER-α,

*© 2001 Cancer Research Campaign*

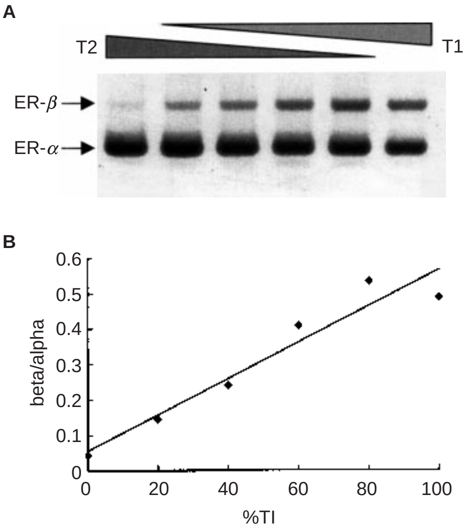

**Figure 1** Multiplex amplification of T1 (moderate ER-β/high ER-α content) and T2 (low ER-β/high ER-α content) cDNA mixed preparations. (**A**) Increasing amounts of T1 cDNA and decreasing amounts of T2 cDNA were mixed and amplified by PCR using ER-α and ER-β specific primers in a single tube. (**B**) PCR products were separated on 1.8% metaphor agarose gels and stained with ethidium bromide

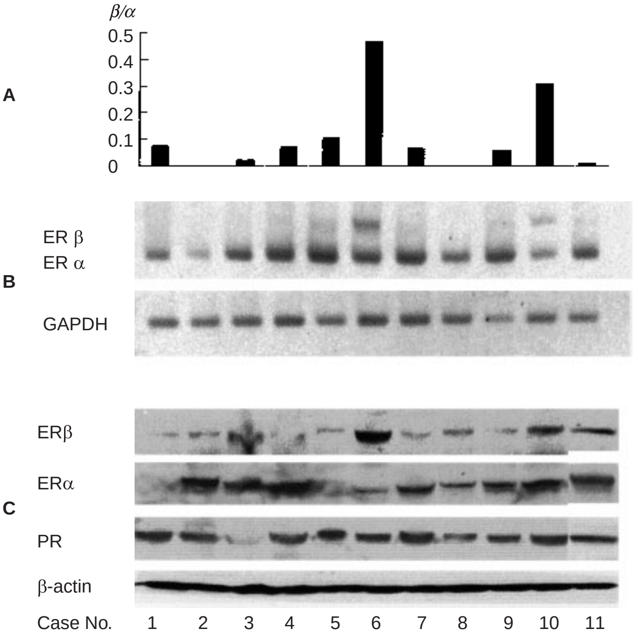

**Figure 2** (**A**) The ratio of ER-β/ER-α mRNA expression in multiplex RT-PCR. (**B**) Multiplex RT-PCR analysis of ER-α and ER-β expression. (**C**) Western blotting analysis of ER-α, ER-β and PR protein. The relative expression of ER-β and ER-α protein were almost correlated with analysis of mRNA. PR protein was expressed in all samples to varying degrees. The case No. is concordant with that of Table 2

and that both ER-α positive (T-47D, T-47D-5) and negative (MDA MB231, MCF 10A1) cell lines express ER-β. They suggested that ER-β expression plays a possible role in human breast cancer. Leygue et al (1998) used multiplex-PCR and a ligand binding assay to show that the ER-α:ER-β ratio significantly increases in tumour components compared with normal components. Therefore, this ratio might play a role in the alteration of oestrogen action that occurs during the development of human breast cancer. In addition, Speirs et al (1999) showed that most breast tumours express ER-β, either alone or in combination with ER-α, and that those tumours coexpressing ER-α and ER-β were node positive and tended to be of higher grade. Thus, the clinical importance of measuring ER-β levels in breast cancer has been established. However, the relevance to endometrial cancer has not been determined.

We are the first to study ER-α and ER-β mRNA coexpression in human endometrial cancer tissue using multiplex RT-PCR and to demonstrate a correlation between the ER-β:ER-α ratio and clinicopathological parameters. We found that ER-α mRNA was expressed in all endometrial carcinomas examined. While the expression of ER-β mRNA varied among tumours and the level of ER-β mRNA was relatively high especially in advanced invasive carcinomas, the ER-β:ER-α mRNA ratio significantly correlated with the depth of myometrial invasion. Western blotting analysis also showed that ER-β proteins were highly expressed in comparison with ER-α proteins in endometrial cancer with severe myometrial invasion. The depth of myometrial invasion is a potent risk factor for endometrial cancer and positively correlates with prognosis (Le Vacchia et al, 1983; Morrow et al, 1991). Therefore, preoperative knowledge of the precise depth of myometrial invasion undoubtedly benefits surgical planning. Ultrasound and MRI imaging or measurement of serum CA125 levels are useful, but when endometriosis or leiomyoma are complicating factors, such findings are not sufficient. The results of the present study demonstrated that understanding the ER-β:ER-α ratio could be beneficial for predicting the depth of myometrial invasion.

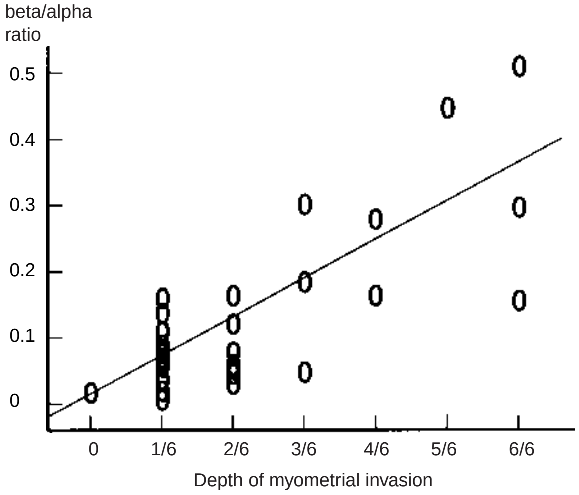

**Figure 3** Analysis of the correlation between ER-β:ER-α ratio and depth of myometrial invasion. Depth of myometrial invasion was categorized into 7 groups, from 0: no invasion to 6/6: reached the serosa. The correlation coefficient was 0.786 ($P < 0.0001$). The association was assessed by Spearman's rank correlation coefficient. *P* values were the result of two-sided test (rS = 0.634 and $t = 4635$ (df = 32)).

The mechanisms behind how the expression of ER-β causes deep endometrial invasion are difficult to define. ER subtypes can signal not only from the classical oestrogen response element but also from an AP1 enhancer element (Gaub et al, 1990; Paech et al, 1997). Therefore, ER-β may elicit its own signals and interfere with those of ER-α. Theoretically, the growth of ER positive cells can be inhibited by antioestrogens, such as tamoxifen or the pure antioestrogen, ICI 182780. Tamoxifen is currently the first-line therapy for treatment of ER positive breast cancer (Osborne et al,

 *© 2001 Cancer Research Campaign*

**Table 3** Multiple regression analysis of factors related to ER-β:ER-α ratio

| | Coefficient | Standard error | *P* value |
|---|---|---|---|
| Age | – 0.003188 | 0.0193 | 0.8706 |
| Tumour grade | – 0.015132 | 0.2497 | 0.9521 |
| Clinical stage | – 0.158564 | 0.1362 | 0.2548 |
| Myometrial invasion | 0.542333 | 0.1419 | 0.0007 |

$R^2$ = 0.4178.

1998). However, some patients who initially respond to tamoxifen eventually go into relapse, probably due to the acquisition of antioestrogen resistance. ER-β positivity tends to be associated with more poorly differentiated breast cancers (Speirs et al, 1999) and it has been shown that ER-β is overexpressed in tamoxifen resistant breast tumour (Speirs et al, 1999). Therefore, the coexpression of ER subtypes in the same tumour may modify the response to oestrogen and the tumour may become deeply invasive even in a low oestrogen environment. On the other hand, tamoxifen acts as an antioestrogenic and as an oestrogen-like substance on the endometrium (Hyder et al, 1996; Berliere et al, 1998), but its molecular mechanism has not yet been confirmed. The expression of ER-α and ER-β may decide the nature of the endometrial cell response to tamoxifen.

Many studies have suggested that the progesterone receptor (PR)/ER concentration is significant as a prognostic parameter for endometrial cancer (Friberg and Noren, 1993; Kadar et al, 1993; Morris et al, 1995; Moutsatsou and Sekeris 1997), and that PR status significantly predicts the disease-free survival of patients with endometrial cancer. It has been shown that lymph node metastasis from endometrial cancer is correlated with negative PR protein expression (Iwai et al, 1999). Therefore, the effect of ER-β on the expression of PR as well as the mechanism of how ER-β affects the nature of endometrial cancer should be clarified. Based on our result, PR protein expression appeared not to be associated with that of ER subtypes and clinicopathological parameters. Only one sample was node positive in our study. Thus, this issue needs to be investigated in studies involving more cases with nodal involvement.

We reported that ER-β/ER-α expression ratio was useful as a novel prognostic indicator. The patients in this study were followed for a limited duration after operation, and thus whether or not ER-β status actually indicates disease-free survival must be established.

In conclusion, although the biochemical function of ER-β in relation to endometrial cancer remains unknown, our results suggest that ER-β plays an important role in the progress of myometrial invasion. If so, ER-β may be an useful prognostic tool for patients with endometrial carcinoma.

### ACKNOWLEDGEMENTS

This work was partially supported by Grant 09671664 from the Ministry of Education, Science, Sports and Culture of Japan.

### REFERENCES

Berliere M, Charles A, Galant C and Donnez J (1998) Uterine side effects of tamoxifen: a need for systematic pretreatment screening. *Obstet Gynecol* **91**: 40–44

Dotzlaw H, Leygue E, Watson P and Murphy L (1997) Expression of estrogen receptor-β in human breast tumors. *J Clin Endocrinol Metab* **82**: 2371–2374

Dotzlaw H, Leygue E, Watson P and Murphy LC (1999) Estrogen receptor-β messenger RNA expression in human breast tumour biopsies: relationship to steroid receptor status and regulation by progestins. *Cancer Res* **59**: 529–532

Friberg LG and Noren H (1993) Prognostic value of steroid hormone receptors for 5-year survival in stage II endometrial cancer. *Cancer* **71**: 3570–3574

Gaub MP, Bellard M, Scheuer I, Chambon P and Sassone CP (1990) Activation of the ovalbumin gene by the estrogen receptor involves the fos-jun complex. *Cell* **63**: 1267–1276

Hyder SM, Stancel GM, Chiapetta C, Murthy L, Boettger THL and Makela S (1996) Uterine expression of vascular endothelial growth factor is increased by estradiol and tamoxifen. *Cancer Res* **56**: 3954–3960

Iwai K, Fukuda K, Hachisuga T, Mori M, Uchiyama M, Iwasaka T and Sugimori H (1999) Prognostic significance of progesterone receptor immunohistochemistry for lymph node metastases in endometrial carcinoma. *Gynecol Oncol* **72**: 351–359

Kadar N, Malfetano JH and Homesley HD (1993) Steroid receptor concentrations in endometrial carcinoma: effect on survival in surgically staged patients. *Gynecol Oncol* **50**: 281–286

Kuiper GGJM, Enmark E, Pelto-Huikko M, Nilsson S and Gustafsson JA (1996) Cloning of a novel estrogen receptor expressed in rat prostate and ovary. *Proc Natl Acad Sci* USA **93**: 5925–5930

Kuiper GGJM, Carlsson B, Grandien K, Enmark E, Haggblad J, Nilsson S and Gustafsson JA (1997) Comparison of the ligand binding specificity and transcript tissue distribution of estrogen receptors alpha and beta. *Endocrinology* **183**: 863–870

La Vecchia C, Franceschi S, Parazzini F Colombo E, Colombo F, Liberat A and Mangioni C (1983) Ten-year survival in 290 patients with endometrial cancer: prognostic factors and therapeutic approach. *Br J Obstet Gynecol* **90**: 654–661

Leygue E, Dotzlaw H, Watson P and Murphy L (1998) Altered estrogen receptor α and β mRNA expression during human breast tumorigenesis. *Cancer Res* **58**: 3197–3201

Lu B, Leygue E, Dotzlaw H, Murphy LJ, Murphy LC and Watson PH (1998) Estrogen receptor-β mRNA variants in human and murine tissues. *Mol Cell Endocrinol* **138**: 199–203

Morris PC, Anderson JR, Anderson B and Bulle RE (1995) Steroid hormone receptor content and lymph node status in endometrial cancer. *Gynecol Oncol* **56**: 406–411

Morrow CP, Bundy BN, Kurman RJ, Creasman WT, Heller P, Homesley HD and Graham JE (1991) Relationship between surgical-pathological risk factors and outcome in clinical stage I and II carcinoma of the endometrium. A Gynecologic Oncology Group Study. *Gynecol Oncol* **40**: 55–65

Mosselman S, Polman J and Dijkema R (1996) ER-β: identification and characterization of a novel estrogen receptor. *FEBS Lett* **392**: 49–53

Moutsatsou P and Sekeris CE (1997) Estrogen and progesterone receptors in the endometrium. *Ann N-Y-Acad Sci* **816**: 99–115

Osborne CK (1998) Tamoxifen in the treatment of breast cancer. *N Engl J Med* **339**: 1609–1618

Paech K, Webb P, Kuiper GGJM, Nilsson S, Gustaffson JA, Kushner PJ and Scanlan TS (1998) Differential ligand activation of estrogen receptors ERα and ERβ at AP-1 sites. *Science* **277**: 1508–1510

Speirs V, Parkes Alicia T, Kerin MJ, Walton DS, Carleton PJ, Fox JN and Atkin SL (1999a) Coexpression of estrogen receptor α and β: poor prognostic factors in human breast cancer? *Cancer Res* **59**: 525–528

Speirs V, Malone C, Walton DS, Kerin MJ and Atkin SL (1999b) Increased expression of estrogen receptor beta mRNA in tamoxifen-resistant breast cancer patients. *Cancer Res* **59**: 5421–5424

Tremblay GB, Tremblay A, Copeland NG, Gilbert D, Jenkins NA, Labrie F and Giguere V (1997) Cloning, chromosomal localization, and functional analysis of the murine estrogen receptor β. *Mol Endocrinol* **11**: 353–365

© 2001 Cancer Research Campaign 
